# Itraconazole and Difluorinated-Curcumin Containing Chitosan Nanoparticle Loaded Hydrogel for Amelioration of Onychomycosis

**DOI:** 10.3390/biomimetics7040206

**Published:** 2022-11-21

**Authors:** Prashant Kesharwani, Mahak Fatima, Vanshikha Singh, Afsana Sheikh, Waleed H. Almalki, Virendra Gajbhiye, Amirhossein Sahebkar

**Affiliations:** 1 Department of Pharmaceutics, School of Pharmaceutical Education and Research, Jamia Hamdard, New Delhi 110062, India; 2 Center for Transdisciplinary Research, Department of Pharmacology, Saveetha Dental College, Saveetha Institute of Medical and Technical Science, Chennai 600077, India; 3 Department of Pharmacology and Toxicology, Faculty of Pharmacy, Umm Al-Qura University, Makkah 24381, Saudi Arabia; 4 Nanobioscience, Agharkar Research Institute, Pune 411004, India; 5 Applied Biomedical Research Center, Mashhad University of Medical Sciences, Mashhad 13944-91388, Iran

**Keywords:** onychomycosis, *Trichophyton mentagrophytes*, chitosan nanoparticle, hydrogel, itraconazole, difluorinated curcumin

## Abstract

Onychomycosis is a nail infection caused by a fungus, *Trichophyton mentagrophytes,* that is responsible for major nail infections. The best method suited for treating such infections generally includes a topical remedy. However, conventional oral or topical formulations are associated with various limitations. Therefore, a more efficient and compatible formulation is developed in this study. The primary objective of the current study is to formulate and evaluate chitosan nanoparticle-based hydrogel for ameliorating onychomycosis. The sole purpose of this research was to increase the permeation of the lipophilic drug itraconazole and difluorinated curcumin, and its synergistic antifungal activity was also evaluated for the first time. Both in vitro and ex vivo drug release evaluations confirmed the sustained release of both drugs from the hydrogel, which is a prerequisite for treating onychomycosis. The results overall highlighted the promising activity of a synergistic approach that could be implemented for the treatment of onychomycosis. The hydrogel-based formulation serves as an effective method of delivery of drugs across the layers of the skin, resulting from its hydrating characteristics.

## 1. Introduction

The human nail plate is one of the toughest protective barriers of the human body. It consists of rigid keratin, which is aligned transversely within the nail plate and is responsible for imparting hardness. Onychomycosis is a chronic fungal infection that develops in fingernails or toenails and affects around 19% of the population worldwide [1]. It might involve infection in any part of the nail, be it the plate area, matrix, or bed [2]. Onychomycosis is generally caused as a result of infection from any of these micro-organisms, including *Trichophyton mentagrophytes*, *Trichophyton rubrum,* or *Candida albicans* [3]. The risk factors associated with the development of this infection include diabetes, smoking, HIV-AIDS, and peripheral vascular arterial disease. The symptoms characterizing this nail infection include roughening, splitting, thickening, the occurrence of irregular surface, or any kind of discoloration or, at times, detachment of the nail plate from the nail bed. The nail keratin in this condition is largely increased which restricts the entry and delivery of drug at the desired topical site of treatment [4]. Recently, scientists developed a topical antifungal formulation using efficonazole as a 10% conventional solution to treat this infection [5]. However, it failed to show maximum effectiveness since the permeability of the drug within the nail was highly reduced, which in turn resulted in demand for higher drug concentration. In addition to this, when applied to the nail surface, the drug solution can be wiped out easily, which eventually reduces its activity. Therefore, a delivery system that can help overcome these issues and can also serve as a positive treatment solution for onychomycosis needs to be devised [6].

In recent years, various studies related to nanoparticle-based drug delivery systems have been brought to use to increase the permeation of drugs at the targeted infectious site [7]. Antifungal drugs like Itraconazole (ITZ) are largely used in nail infections due to their high efficacy and lower side effects [8]. Similarly, Difluorinated curcumin (CDF) can also be utilized for its antifungal properties.

Considering these drugs are highly expensive, and for them to show high efficacy, a sufficient concentration would be needed. The most convenient form of topical treatment for nail infection is the use of nail lacquer. The organic component present in it evaporates to leave a reliable layer on the surface. This film serves as a drug depot for a longer intended time [9]. Additionally, nano-based drug delivery is brought into focus that not only helps reduce the concentration required but also enhances the permeability of the drug across nail plate, is versatile in nature, and is biocompatible, as well as a longer shelf life in comparison to conventional formulations [10]. The nanoformulations that are being utilized include nanoparticles, nanoemulsions, liposomes [11], dendrimers, nanogels, nanovesicles, etc. [12]. In one of the studies performed by scientists, an oil-in-water microemulsion of itraconazole and terbinafine was prepared for the treatment of onychomycosis [13]. The formulated microemulsion showed effectivity in a much lower concentration of drug in comparison to the conventional formulation. [14]. Hydrogels are another topical formulation that is largely popular against infections and antifungal therapy [15] owing to their biocompatibility, water-holding capacity, and soft nature. They are formed by the physical or chemical cross-linking of polymers. At times, smart polymer with thermo-responsive characteristics is also utilized to ensure sol-to-gel transition with changing temperatures [16,17].

Under similar lines, in this experiment, we have formulated, optimized, and evaluated nanoparticles loaded with a hydrogel that not only enhanced permeation but also enhanced residence time and provide good release of the drug for a longer time period. ITZ and CDF are utilized for loading within the nanoparticles. Nanoparticles were prepared utilizing chitosan. Chitosan is a natural polymer obtained from crustacean shells of crabs, prawns, and at times from fungal cell walls. It is highly biocompatible, biodegradable, mucoadhesive, and safe for topical use. Chitosan is formed as a result of partial deacetylation of N-acetyl glucosamine obtained after the alkaline deacetylation of chitin. It comprises a β-(1,4)-linked-D-glucosamine residue with the amine groups randomly acetylated. The functional groups, such as the amine and –OH groups, provide chitosan with various different characteristics making it a versatile option to be used in various different areas [18]. Chitosan nanoparticles are largely utilized as vehicles for drug, gene, or protein delivery and are largely used to form complexes like gels [19]. In the current study, both these drugs are antifungal drugs and have high lipophilicity and low binding affinity with keratin. Limited work is available with the use of itraconazole against onychomycosis, while no work has been reported for CDF to date. Herbal phytochemicals such as curcuminoids are largely being brought to use for treatment against infections caused by candida species [20]. This study depicts the synergistic effects of both drugs loaded within nanoparticles. Hydrogel formulation was later prepared to enhance its retention time. It has been reported that nail behaves as hydrogel upon hydrating with an aqueous solution and shows better permeation. The hydration causes the keratin network and disulfide bonds between them to loosen a little, which creates channels for the drugs to pass [21,22]. This study was performed for the sole purpose of facilitating delivery of either lipophilic drugs or drugs that have some solubilization issues. In the current study, drug-loaded nanoparticle hydrogel was compared against free drug or drug-loaded plain hydrogel. Carbopol is utilized as the polymer for the preparation of hydrogel owing to its biocompatibility and enhanced solubilization and permeation [23]. It is largely used in the cosmetic industry as a thickener in lotions, creams, and gels. The activity and efficacy of the prepared formulation were demonstrated through in vitro and ex-vivo permeation studies and nail clipping studies.

## 2. Materials and Methods

### 2.1. Materials

Itraconazole was received as gift samples from CDH fine chemicals, India. Difluorinated curcumin was synthesized as described earlier [24,25]. High molecular weight chitosan, glacial acetic acid, Disodium Hydrogen Phosphate, Sodium Tripolyphosphate (Na-TPP), and Triethanol amine (TEA) were procured from TCI. Carbopol 940, Potassium dihydrogen phosphate, sodium chloride Tween 20, DMSO, ethanol, and methanol were obtained from SD fine, Mumbai, India.

### 2.2. Solubility Analysis and Analytical Method Development

The solubility of both drugs was analyzed in different solvents, and the UV–Visible spectrophotometric method for both drugs, i.e., ITZ and CDF, was developed. The UV–Visible spectrophotometer (Shimadzu-UV-1601, Kyoto, Japan) was set in the range of 200–800 nm to analyze the drug content in the selected solvent at around 270 nm and 453 nm for ITZ and CDF, respectively, for their drug release studies from the prepared formulation [26]

### 2.3. Preparation of Chitosan Nanoparticles by Ionotropic Gelation Technique

To prepare the chitosan NPs, firstly, a solution of glacial acetic acid (5% *v/v*) in deionized water was prepared and 0.1% of high molecular weight chitosan was added it. The solution was left on magnetic stirrer at 37 °C overnight for swelling of the chitosan. Next day, few drops of Tween 20 surfactant were incorporated to the prepared solution, its pH was adjusted to 5–6 using 0.1 N sodium hydroxide. Separate solutions of 1% itraconazole and 0.012% CDF were prepared by dissolving the drugs in DMSO. These were then drop-wise added to the chitosan solution and were left on, stirring for two hours at 1000 rpm. Separately, 0.1% of Na-TPP solution was prepared in distilled water as Na-TPP was used here as cross-linking agent. Na-TPP was then drop-wise added to the solution of chitosan at high stirring until the solution turned colloidal in nature. The solution was centrifuged at 10,000 rpm, 4 °C for 15 min to separate out the NPs. Then the NPs were collected and were further washed with ultrapure water and later lyophilized to yield ITZ-CDF/CH NPs. The prepared formulation was then subjected to distinctive characterization studies [27].

### 2.4. Evaluation of Physiochemical Properties of Drug-Loaded Chitosan Nanoparticles 

#### 2.4.1. Particle Size, Polydispersity Index (PDI), and Zeta Potential

The size of the prepared NPs, their PDI, and surface charge was analyzed using Malvern Zetasizer. For analysis, firstly, the formulation has to be diluted with distilled water, then this diluted preparation would be transferred to a quartz cuvette of the zetasizer for size, PDI, and surface charge analysis (*n* = 3) [28].

#### 2.4.2. Morphological Examination via Transmission Electron Microscopy

For determination of the surface structure of the chitosan NPs, the formulation was subjected to transmission electron microscopy (TEM) (HR-TEM FEI, TECNAI G2 {200 kV}, The Netherlands). The sample was diluted and kept on a copper grid, followed by staining with 2% phosphotungstic acid aqueous solution and was allowed to dry for 30 s. The grid was then scanned under a microscope.

#### 2.4.3. Entrapment Efficiency and Drug Loading Determination

The entrapment efficiency and drug loading were analyzed to calculate the entrapment ability of chitosan nanoparticles and the amount of ITZ and CDF loaded in the prepared nanoparticles. The required amount of ITZ-CDF/CH NPs was dispersed in PBS: methanol solution (7:3) and centrifuged for 30 min at 15,000 rpm. The supernatant was withdrawn, and the un-entrapped amount of drug in nanoparticles was evaluated after further dilution and absorbance measurement of the withdrawn supernatant with the help of a UV-spectrophotometer. The given equation was used to calculate %EE and %DL:

% EE = Total drug added − Total free drug/Total drug added × 100(1)

% DL = Amount of total entrapped drug/Weight of total nanoparticles × 100(2)

#### 2.4.4. Differential Scanning Calorimetry (DSC) 

DSC analysis was done using Perkin Elmer (Rodgau, Germany) instrument. For this, approximately 8–10 mg of lyophilized formulation ITZ-CDF/CH NPs was placed inside aluminium pans that were sealed hermetically. The thermo-grams were then recorded between the temperature range of 30–400 °C, and the speed of heating and cooling was around 10 °C/min [29].

#### 2.4.5. Fourier Transform Infrared Spectroscopy (FT-IR)

Similar to DSC, IR spectra of ITZ-CDF/CH NPs were observed. The potassium bromide pellets of all the samples were prepared and then analyzed in a range of 400–4000 cm^−1^ using Perkin Elmer (Rodgau, Germany) spectrophotometer [29].

### 2.5. Preparation of ITZ-CDF/CH NPs Loaded Hydrogel

Hydrogel preparation was done by direct dispersion method. In this method, carbopol 940 was used as a gelling agent. Carbopol was added to warm water (40 °C) while stirring continuously at 500 rpm for 30 min. Once dissolved completely, the solution was kept overnight at 25 °C. 1 mg of drug-loaded nanoparticles dissolved in a minimum quantity of water was later added to this carbopol solution, and the pH of the gel was adjusted by TEA while placing it under continuous stirring of 500 rpm. Continuous stirring resulted in the formation of homogenous hydrogel without the formation of lumps [30].

### 2.6. Evaluation of Physiochemical Properties of Hydrogel

#### 2.6.1. pH Determination

Any alterations in pH values of a formulation affect the ionization of drugs (be it acidic or basic) and indirectly influence nail permeability. As reported, the pH of the nail is 4.8–5.4. The pH of the prepared formulation was calculated using the pH meter.

#### 2.6.2. Spreadability Study

The spreading ability of the NPs containing hydrogel was determined by taking two glass slides of size around 20 cm × 20 cm and placing a minimum quantity of the formulation in the center of one of the slides. A second glass slide was then kept on top of it and was subjected to a weight of 1000 g for 1 min, resulting in the spreading of gel between the 2 slides. The diameter of the gel spread was determined. The results of the study were obtained in the form of triplicates [31].

#### 2.6.3. Extrudability Study

Upon application of certain weight, gel extrudes from the tube, to assess the quantity of gel extruded, extrudability study was conducted. To calculate the extrudability, the following formula is used:

Extrudability = Force applied to extrude gel from the tube

Cross-sectional area of the syringe

For optimum extrudability, in 10 s, at least 0.5 cm of ribbon gel should be extruded [32].

#### 2.6.4. Viscosity and Texture 

Brookfield viscometer was used to measure the viscosity of hydrogel. Spindle number 3 of the viscometer at a speed of 20 rpm/min was run to analyze the viscous nature of the gel. Firstly, the spindle was properly positioned. The instrument was read at the end of ten minutes. Alongside this texture of the hydrogel was measured using the texture analyzer (TA.XT Plus, Texture Technologies, New York, NY, USA).

### 2.7. In Vitro Drug Release Study 

The NPs containing hydrogel formulation and drug containing plain hydrogel formulation was subjected to a comparative in vitro drug release study to determine the amount of drug released from both formulations. A 0.8 μm pore-sized dialysis membrane (membrane no. 60, Himedia^®^, Mumbai, India) was selected for this study because of its biocompatible nature and suitable porosity index. The use of this membrane in evaluating drug release of different nail formulations in vitro is already established. The membrane was initially activated by a series of steps which primarily involved washing the membrane for 5–6 h under running water. Later, it was rinsed with 0.3% sodium sulfide solution for 60 s and then soaked in hot water of temperature 70–80 °C for about 3–4 min. The membrane was then acidified by treating it for 2–3 min with 0.2% sulphuric acid and washing it again with hot water for 3–4 min. Lastly, the membrane was soaked overnight in PBS solution with a pH maintained around 7.2 [33].

The membrane was then carefully mounted on the diameter of the Franz diffusion cell, keeping in check for the presence of any air bubbles. The receptor compartment of the cell was already filled with solvent PBS: methanol (7:3), 1 g drug containing plain hydrogel, and ITZ-CDF/CH NPs loaded hydrogel was added to the donor compartment, and this setup was then kept at 100 rpm for 24 h, temperature maintained at 37 ± 2 °C. All the openings were sealed with parafilm to prevent loss by evaporation. A sample of 0.9 mL was taken out at regular intervals of 0, 1, 2, 3, 4, 6, 8, 12, and 24 h, and sink conditions were maintained by immediately adding the same amount of solvent back. Lastly, the absorbance was checked using a UV spectrophotometer [34].

### 2.8. Transungual Permeation Study

Hoof membrane from bovine was freshly collected from the local butchery. Bovine membrane resembles human nail plates, and upon incubation with keratinase, it releases keratin protein. Thus, it is prominently used in nail permeation studies [35]. Prior to use, the membrane was soaked in PBS pH 7.4 for 24 h [36].

The membrane was then carefully mounted on the diameter of the Franz cell. PBS: methanol (7:3) was filled in the receptor compartment of the cell, and 1 g of drug containing plain hydrogel and ITZ-CDF/CH NPs loaded hydrogel was added to the donor compartment. This setup was then kept at 100 rpm for 24 h, temperature maintained at 37 ± 2 °C. All the openings were sealed with parafilm to prevent loss by evaporation. 0.9 mL sample was taken out at regular intervals of 0, 1, 2, 4, 6, 8, 12, and 24 h, and sink conditions were maintained by immediately adding the same amount of solvent back. Lastly, the absorbance was checked using a UV spectrophotometer [37].

### 2.9. Nail Clipping Study

Nails of healthy human volunteers were collected for performing this study. Keeping in check that no ethical approval is needed for nails since nails are waste material and can be donated, yet after giving a detailed explanation about the study to the patients and getting consent from individual volunteers, nails were collected from the hand or toe. Toenail or hand nail clippings of length around 3.29 mm, width around 2.81 mm, and thickness around 0.32 mm for the age group between 20 and 30 years were collected, and the study was conducted in groups of three (Free ITZ, free CDF, and ITZ-CDF/CH NPs) in triplicate. The volunteers were restricted from consuming any kind of drug/medicine during the time of nail collection. The nails obtained were washed with water and later dried after wiping them with tissue paper. They were kept in a Petri plate and stored at 25 °C until use. In 9 different 2 mL glass vials, a volunteer nail sample was added. To the first triplicate, ITZ-CDF/CH NPs solution was added, while to the other six, free ITZ and free CDF were added. The glass vials were sealed and kept overnight. Nail digestion that occurred during this time period was used to determine drug loading within the nail plates. Nails were later removed and then wiped with damp cotton and finally with a clean tissue. These samples were then soaked in 1 M of NaOH and kept on stirring for 24 h. It was later acidified using 1M HCl to form a neutralized solution. After this, centrifugation was performed by adding PBS: methanol (7:3) to each solution, and the collected residue was dried. The residue was then washed with 2 mL PBS: methanol (7:3) and preserved as a solution which was then analyzed using UV Spectrophotometer [5].

### 2.10. Antifungal Activity

In vitro antifungal activity was determined using the cylinder plate method, and the fungal strain used was *Trichophyton mentagrophytes.* Four formulations, i.e., blank nanoparticles, free ITZ, free CDF, and ITZ-CDF/CH NPs, were analyzed for their antifungal action. The fungal strain was cultivated in sabouraud dextrose agar media for 7 days at 25 °C. The harvested spores were then added into 20 mL media and then filtered by passing them through sterile gauze. Sabouraud dextrose agar (100 mL) was taken as growth media, and inoculated media was then added to it and maintained at 37 ± 1 °C temperature. Stock solutions of ITZ, CDF, and ITZ-CDF/CH NPs were prepared, and then required dilutions (50, 100, 150 μg/mL) were prepared from stock. Three wells were bored in each Petri dish, and 0.1 g of all three dilutions from three formulations were filled in the wells. Then these dishes were incubated at 25 °C for a week. Colony forming unit (CFU) and log CFU was then calculated, recorded, and compared for all formulations [38].

## 3. Results and Discussion 

### 3.1. Solubility Analysis and Analytical Method Development

The solubility study of ITZ and CDF was carried out in different solvent mediums, and it was noted that they both were insoluble in water, HCl, and PBS solution of different pH but completely solubilized in DMSO and methanol.

A UV analytical method for ITZ and CDF was developed. R^2^ (Regression coefficient) for ITZ for 2–10 g/mL concentration range with solvent PBS: methanol (7:3) was 0.9991 at 270 nm, and R^2^ of CDF in a concentration range of 0.1–1 g/mL in the same solvent medium was 0.998 at 453 nm.

### 3.2. Preparation of Chitosan Nanoparticles by Ionotropic Gelation Technique

**Figure 1:**
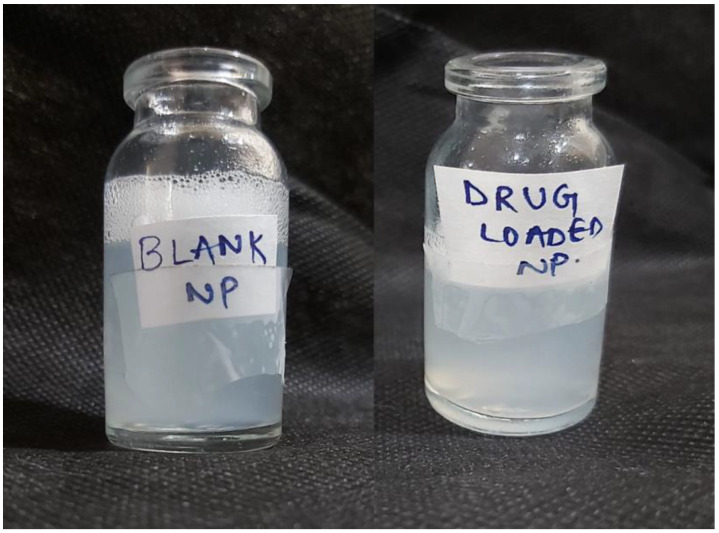
Blank and drug-loaded chitosan nanoparticles.

The chitosan nanoparticles (ITZ-CDF/CH NPs) were prepared (Figure 1) and lyophilized to enhance their stability and to use for different evaluation studies.

### 3.3. Evaluation of Physiochemical Properties of Drug-Loaded Chitosan Nanoparticles

#### 3.3.1. Particle Size, Polydispersity Index (PDI), and Zeta Potential Analysis

**Figure 2:**
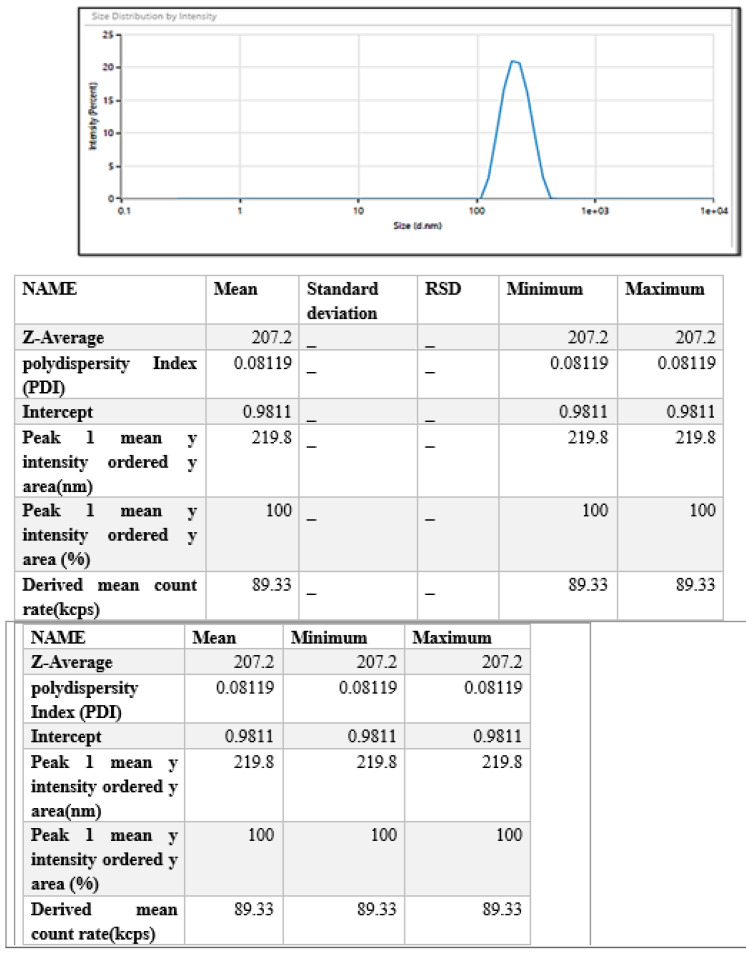
Particle size and polydispersity index of drug-loaded chitosan nanoparticles (ITZ-CDF/CH NPs).

The size of the prepared NPs, their PDI, and surface charge was analyzed, and particle size was noted to be 207.2 nm which is a prerequisite for chitosan nanoparticles as this size range shows excellent permeability into deeper layers of the skin [39], while the PDI was noted to be around 0.08, corresponding to the uniform particle dispersion in the system (Figure 2).

**Figure 3:**
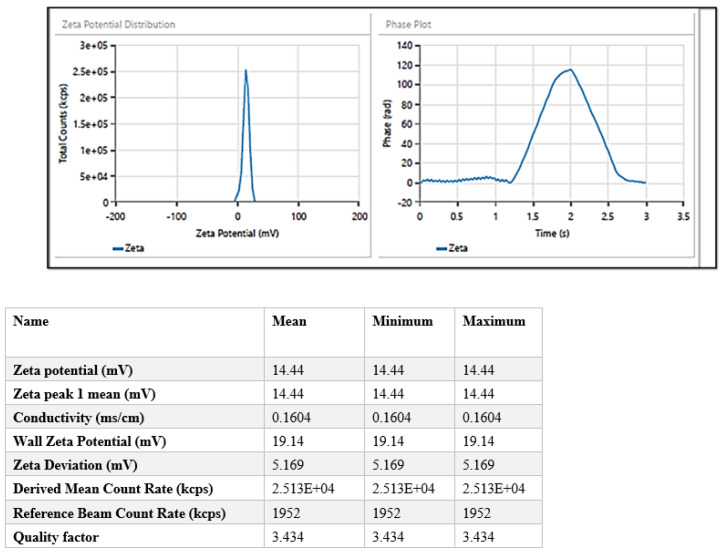
Zeta potential of drug-loaded chitosan nanoparticles (ITZ-CDF/CH NPs).

Zeta potential or surface charge evaluates the stability of the dispersed system. It reveals the extent of particle coagulation occurring as a result of electrostatic repulsion. For formulating NPs, non-ionic surfactants are generally preferred as they limit toxicity and promote compatibility and stability converse to amphoteric or ionic surfactants [40]. The lyophilized nanoparticles showed a zeta potential of 14.44 ± 3.74 mV indicating the presence of chitosan on the surface (Figure 3).

#### 3.3.2. Morphological Examination via Transmission Electron Microscopy

**Figure 4:**
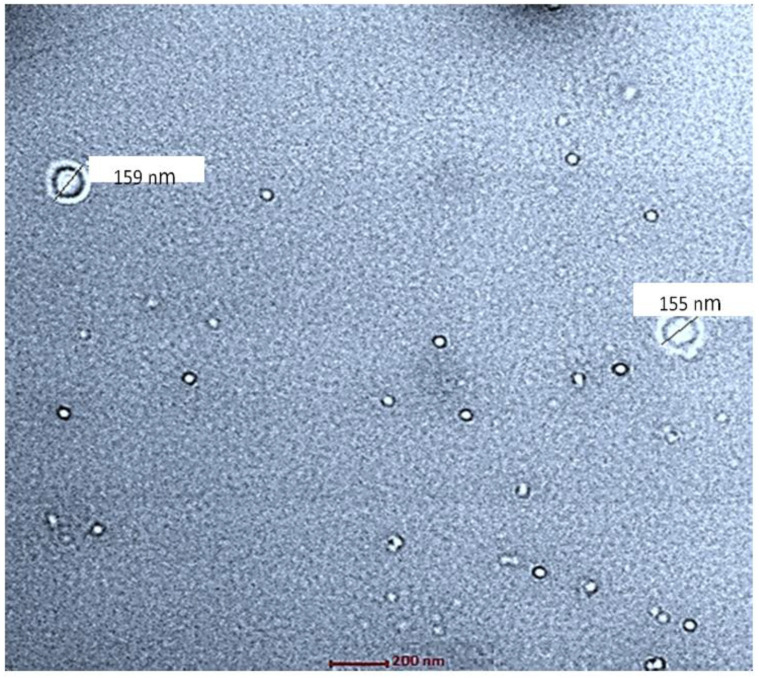
TEM analysis of drug-loaded chitosan nanoparticles (ITZ-CDF/CH NPs).

The spherical structure of the NPs was confirmed via TEM analysis. The results of TEM also show the size of NPs in the range of 100–200 nm (Figure 4).

#### 3.3.3. Entrapment Efficiency and Drug Loading Determination

Drug content and entrapment efficiency are used to estimate the quantity of drug present in the formulation. The drug loading percentage was calculated to be around 15.194% and 8.06% (±) for ITZ and CDF, respectively, while the encapsulation efficiency was calculated to be around 61.4% combined for both drugs.

#### 3.3.4. Differential Scanning Calorimetry (DSC)

**Figure 5:**
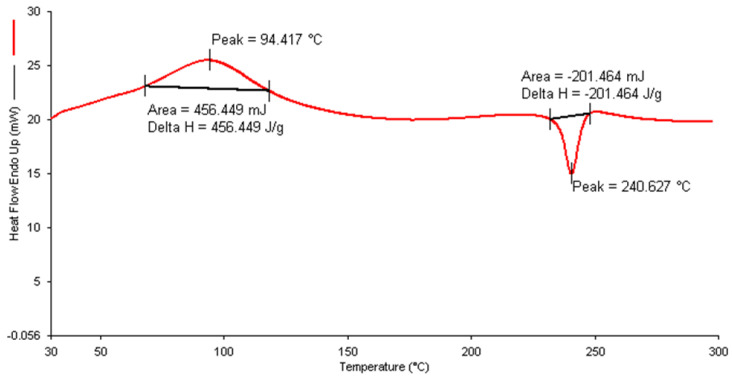
DSC thermogram of drug-loaded chitosan nanoparticles (ITZ-CDF/CH NPs).

The DSC thermogram of both ITZ and CDF shows a sharp endothermic peak at 165.89 °C and 220 °C, respectively [25,41], whereas chitosan exhibit a broad exothermic peak at 299.2 °C [42]. The thermogram of the prepared formulation ITZ-CDF/CH NPs, when observed, showed the absence of ITZ and CDF peaks, while a broad endothermic peak at 94.41 °C and a new exothermic peak at 240.62 °C was detected on the thermogram (Figure 5). The broad endothermic peak at 94.41 °C represents the release of moisture/water molecules present in the formulation ITZ-CDF/CH NPs during the heating process.

The shift in the exothermic peak of chitosan from 299.2 °C to 240.62 °C is probably because of the degradation and disruption of the ionic interaction between the polyanionic TPP molecules and polycationic chitosan. Also, the peak of both drugs does not appear in this graph. This confirms the complete encapsulation of the drug inside the chitosan nanoparticles [43].

#### 3.3.5. Fourier Transform Infrared Spectroscopy (FT-IR)

**Figure 6:**
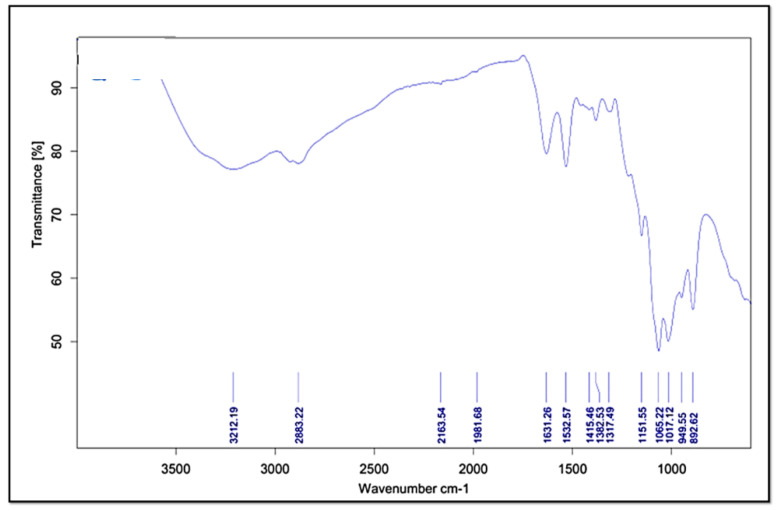
FTIR spectra of drug-loaded chitosan nanoparticles (ITZ-CDF/CH NPs).

The FTIR study was used to explore the structural properties of ITZ-CDF/CH NPs (Figure 6). Precise peaks were detected at 949.55 and 1631.26 cm^−1^, which relate to anhydrous glucosidic and amide (-CONH_2_) of chitosan, respectively (Figure 6). The peaks at 1631 cm^−1^ (CONH_2_) and 1532.57 cm^−1^ (NH_2_) in the spectrum were sharper and represented the presence of redistributed vibrational frequencies induced by chitosan. The minor shifting in the peaks generally depicts the bonds stretching in the sugar ring of the chitosan, and the bonds involved are dipole moments, hydrogen bonds, and van der Waals forces. The shifting in the peaks represents the association of the N-H or O-H bond in the formulation [44]. The peak at 1631.26 cm^−1^ focused on the stretching and vibration between the N-H or O-H group and sugar ring and in the synthesis of ITZ-CDF/CH NPs. It was revealed from the FT-IR spectra that the sugar ring of chitosan contains bonds that may be responsible for encapsulating the drug [45].

### 3.4. Preparation of ITZ-CDF/CH NPs Loaded Hydrogel 

**Figure 7:**
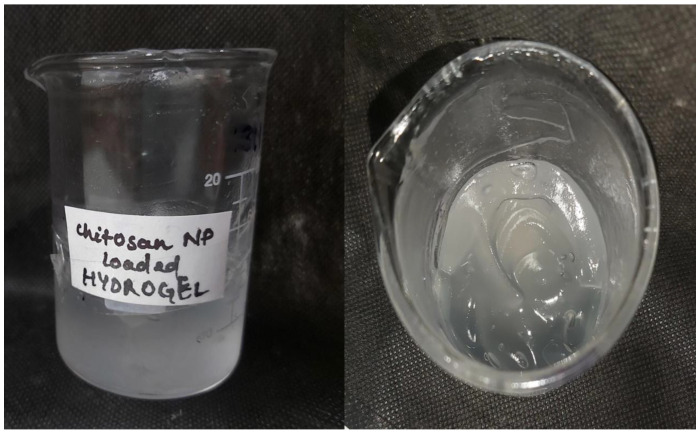
ITZ-CDF/CH NPs loaded hydrogel.

The nanoparticles were prepared using chitosan, whereas the hydrogel formulation was prepared using carbopol (Figure 7). The formulation was viscous, transparent, and homogenous with an acceptable pH value which ensured negligible skin or nail irritation upon application.

### 3.5. Evaluation of Physiochemical Properties of Hydrogel 

#### 3.5.1. pH Determination

The pH value of the optimized formulation was 5.96 ± 0.37, indicating the skin compatibility of the prepared formulation and minimal chances of causing any skin irritation.

#### 3.5.2. Spreadability Study

The spreadability study is done to evaluate the spreading capacity of the hydrogel. The spreadability was noted to be around 3.45 cm for normal weight, 3.55 cm for a weight of 0.5 g, 3.68 cm for 100 g, and 4.56 cm for 1 kg, indicating the easily spreadable nature of the gel.

#### 3.5.3. Extrudability Study

This determines the extrusion of the gel from the tube upon application of precise weight. In 10 s, at least 0.5 cm of ribbon gel should be extruded. Further, the gel having good extrudability extruded optimum gel from the tube [46]. The extrudability of ITZ-CDF/CH NPs loaded hydrogel was found to be 2.1 ± 0.36 gm/cm^2^, which is considered optimal.

#### 3.5.4. Viscosity and Texture 

**Figure 8:**
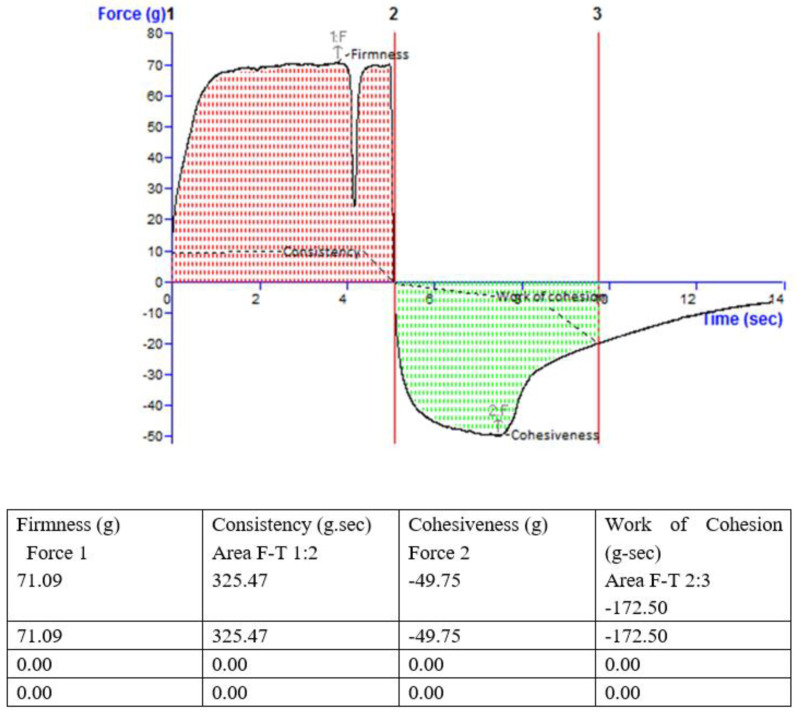
Texture analysis report.

The viscosity of the topical hydrogel measured using a Brookfield viscometer was noted to be around 980.43 cP, and it was noted from the texture analyzer that the formulated hydrogel had the right texture (Figure 8). The mechanical characteristics of the hydrogel formulation include analysis of consistency, firmness, and cohesiveness. These characteristics have a direct effect on the applicability of gel onto the affected surface and patient compliance. The stickiness of gel is determined by Viscosity index, while strength of the gel is indicated by the firmness value. A higher value represents good gel strength [47]. The values of consistency, cohesiveness, firmness, and work of cohesion for prepared hydrogel were found to be 325.47 g/s, −49.75 g, 71.09 g, and −172.50 g/s, respectively.

### 3.6. In Vitro Drug Release Study 

**Figure 9:**
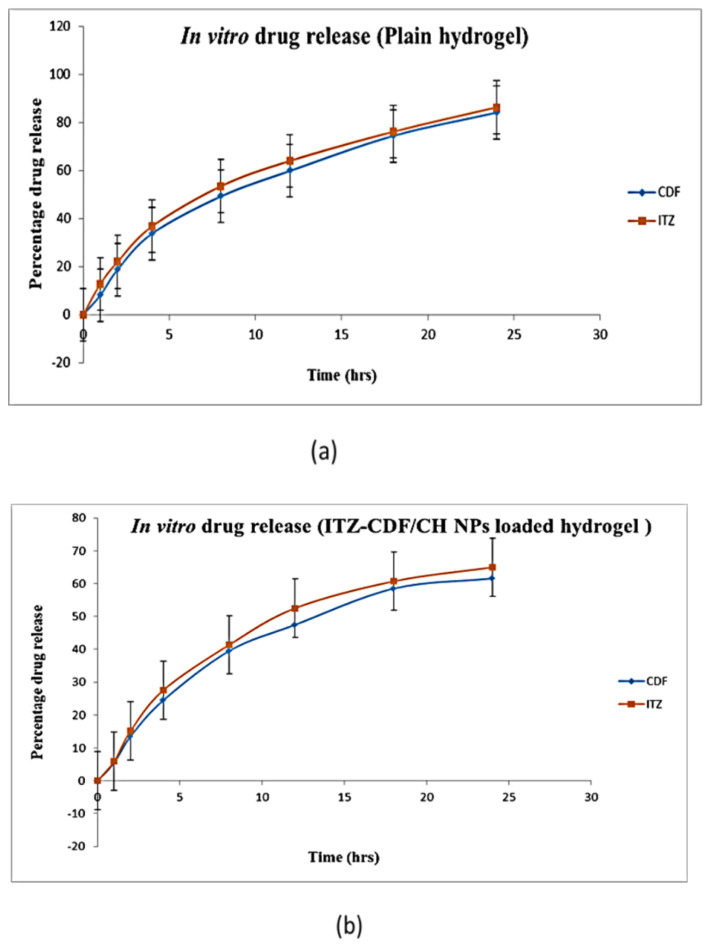
In vitro drug release assessment of (**a**) dual drug-loaded Plain hydrogel and (**b**) ITZ-CDF/CH NPs loaded hydrogel.

The drug release of the drug containing plain hydrogel and ITZ-CDF/CH NPs loaded hydrogel was analyzed for 24 h and the graph between percentage drug release and time was plotted for both the formulations. Quantities of 84.12 ± 0.0045% CDF and 86.3 ± 0.017% ITZ were released from the drug that contained plain hydrogel, while 61.6 ± 0.0017% CDF and 64.98 ± 0.023% ITZ was released from the ITZ-CDF/CH NPs loaded hydrogel (Figure 9). It was observed that in the case of plain hydrogel, both the drugs showed burst release initially, more than 50% of the drug was released in 12 h, and no control drug release pattern was followed till the end of the study. However, a sustained release pattern was observed from the ITZ-CDF/CH NPs loaded hydrogel. The drug’s release was continuous, even after the 12 h, indicating the desired better efficacy of the prepared hydrogel.

### 3.7. Transungual Permeation Study

**Figure 10:**
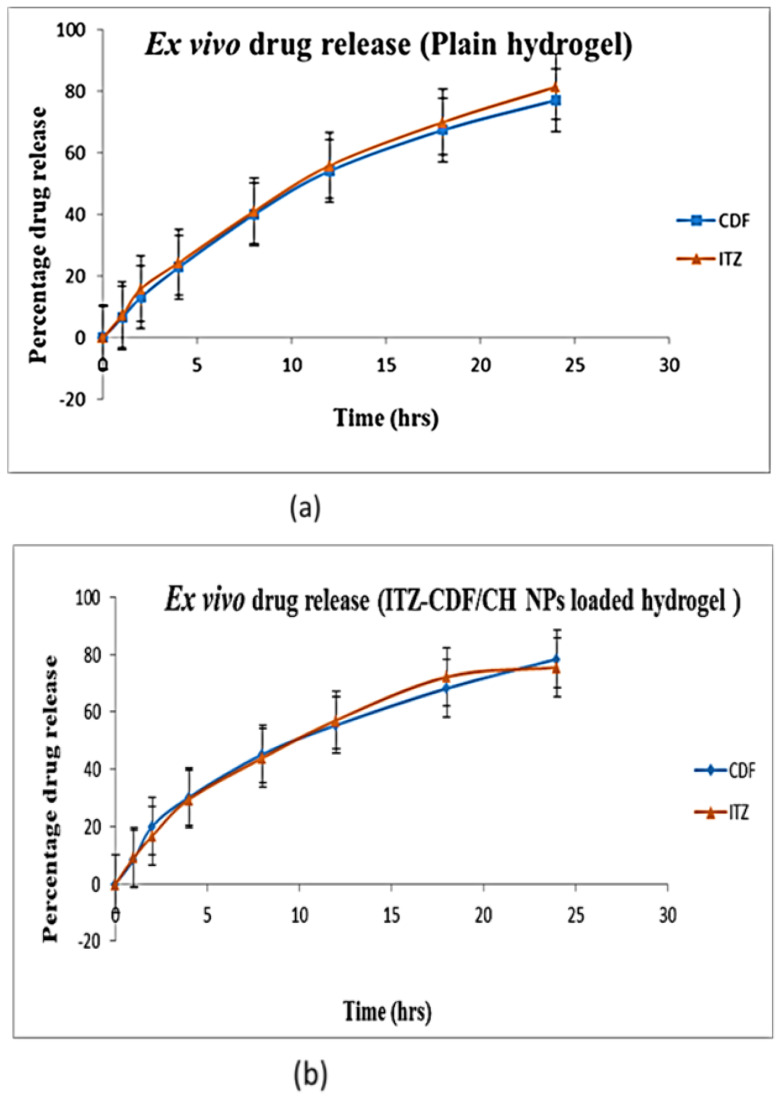
Ex vivo drug release assessment of (**a**) dual drug-loaded Plain hydrogel and (**b**) ITZ-CDF/CH NPs loaded hydrogel.

A transungual permeation study done using a bovine hoof membrane exhibited results similar to in vitro drug release. The drug release from the plain hydrogel exhibited initial burst release and uncontrolled drug release till the end of the study. Contrary to this, ITZ-CDF/CH NPs loaded hydrogel showed sustained drug release at all-time points: 77.06 ± 0.0015% CDF and 81.38 ± 0.04% ITZ was released from the drug that contained plain hydrogel, while 78.38 ± 0.002% CDF and 75.48 ± 0.017% ITZ was released from the ITZ-CDF/CH NPs loaded hydrogel (Figure 10). The sustained release of the drug from the nanoparticle-loaded gel is attributed to the versatile structure of the chitosan nanoparticles that entrapped the drug and released it at a sustained and steady rate. This kind of release is desirable in treating fungal infections as the causative agent gets deep-seated under the nail/skin. Thus, sustained release of the drug leads to their longer antifungal activity, thus causing complete eradication of the fungus [40].

### 3.8. In Vitro Antifungal Activity

**Figure 11:**
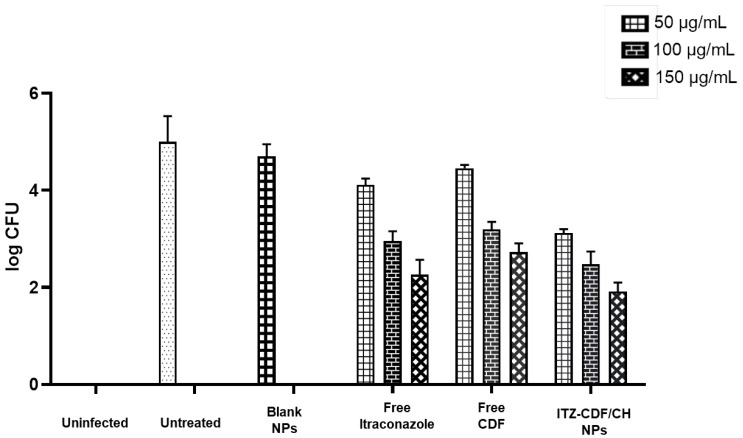
In vitro antifungal activity of free ITZ, free CDF, and ITZ-CDF/CH NPs conducted on *Trichophyton mentagrophytes*, the main causative agent of onychomycosis.

In vitro antifungal activity showed that during the incubation period, free ITZ and free CDF showed almost similar colony-forming units (CFU) (Figure 11). However, when these drugs were encapsulated inside the chitosan NPs to formulate ITZ-CDF/CH NPs, the combined antifungal activity of the formulation enhanced, and CFU decreased. This enhanced activity is attributed to the nanometric size range of formulation, which allows the slow and steady release of drugs from the polymer matrix. As already mentioned, the antifungal action of a drug is a prerequisite for treating onychomycosis, and the developed formulation would accelerate the process of treating the disease.

### 3.9. Nail Clipping Study 

The study depicted that the amount of drug that permeated through the nail in the four samples was calculated to be 1.33 ± 0.02 μg/mg, 0.89 ± 0.08 μg/mg, and 0.85 ± 0.05 μg/mg for ITZ-CDF/CH NPs, free ITZ and free CDF respectively. The drug-loaded nanoparticles must have clearly penetrated the hard nail keratin matrix, owing to their size. Hence, it was concluded that the nanoparticles lead to enhanced penetration of the drug into the layers of the nails as compared to free drugs. The small globule size of the prepared formulation leads to its enhanced penetration into the nails and greater affinity of encapsulated drug towards the keratin present in the nail matrix. Hence, the nail clipping study corresponds with the in vitro as well as ex vivo study results, and it was concluded that after the application of ITZ-CDF/CH NPs, a substantial quantity of drug permeated the nail plate and nail bed [48,49,50].

## 4. Conclusions

In the current study, a synergistic approach with the use of two antifungal drugs was explored to understand their efficacy against onychomycosis. The treatment of such nail infections is slightly difficult, considering it requires that the drug permeates the lower layers of the nail for effective therapy. Therefore, these drugs were loaded within chitosan nanoparticles, which showed good biocompatibility and biodegradability on application. Considering that the residence time plays a major role in the concentration of the drug that is being used, the nanoformulation was made viscous in the form of hydrogel to increase retention over the nail plate. Hydrogels form a film over the nail surface and increase permeation due to hydration which in turn enhances the delivery of the drug and reduces the overall concentration of the drug that needs to be loaded within it [51]. In this study, we have also compared the permeation of drugs from various formulations, including drug-loaded nanoparticles, drugs in free form, and drugs in plain hydrogel. The hydrogel formed a good film over the nail plate and ensured easy application and use. The drug-loaded nanoparticles were found to be stable and showed no signs of degradation. While it can easily be applied, it could also be removed that easily considering it has a gel-like consistency that can be eroded after washing with water under pressure but did not erode in the presence of moisture. The drug release from the chitosan nanoparticles-loaded hydrogel was more sustained than the plain drug-loaded hydrogel, both in vitro and ex vivo. Similarly, the in vitro antifungal activity showed a significant decrease in colony forming units upon application of prepared nanoparticles containing combination of drugs, as compared to free ITZ and free CDF. Overall, the prepared formulation proves to be an effective method of treating onychomycosis and can be a breakthrough for curing different nail infections. Moreover, what cannot be sidelined is that even after years of therapy, the probability that the condition might relapse is still not negligible. Several treatment options are being brought to use, which come with either elevated costs or patients’ dependency on others of some sort.

## Data Availability

Not applicable.
